# Child mental illness and the help-seeking process: a qualitative study among parents in a Ugandan community

**DOI:** 10.1186/s13034-019-0262-7

**Published:** 2019-01-11

**Authors:** V. Skylstad, A. Akol, G. Ndeezi, J. Nalugya, K. M. Moland, J. K. Tumwine, I. M. S. Engebretsen

**Affiliations:** 1 Centre for International Health (CIH), Department of Global Public Health and Primary Care (IGS), University of Bergen, Bergen, Norway; 2 School of Public Health, Makerere University College of Health Sciences, Kampala, Uganda; 3 Department of Psychiatry, Makerere University College of Health Sciences, Kampala, Uganda; 4 Department of Paediatrics and Child Health, Makerere University College of Health Sciences, Kampala, Uganda

## Abstract

**Background:**

Child mental illness contributes significantly to the burden of disease worldwide, and many are left untreated due to factors on both the provider and user side. Recognising this, the Ugandan Ministry of Health recently released the Child and Adolescent Mental Health (CAMH) Policy Guidelines. However, for implementation to be successful the suggested policy changes must resonate with the service users. To better understand the sociocultural factors influencing parental mental help-seeking, we sought insights from parents in the Mbale district of eastern Uganda.

**Method:**

In this qualitative study, eight focus group discussions were conducted with mothers and fathers in urban and rural communities. Parents of children younger than 10 years were purposively selected to discuss a vignette story about a child with symptoms of depression or ADHD as well as general themes relating to child mental illness. The data were analysed using qualitative content analysis.

**Results:**

Descriptions of severe symptoms and epileptic seizures were emphasised when recognising problem behaviour as mental illness, as opposed to mere ‘stubbornness’ or challenging behaviour. A mixture of supernatural, biomedical, and environmental understandings as underlying causes was reflected in the help-seeking process, and different treatment providers and relevant institutions, such as schools, were contacted simultaneously. A notion of weakened community social support structures hampered access to care.

**Conclusion:**

Awareness of symptoms closer to normal behaviour must be increased in order to improve the recognition of common mental illnesses in children. Stakeholders should capitalise on the common recognition of the importance of the school when planning the upscaling of and improved access to services. Multifactorial beliefs within the spiritual and biomedical realms about the causes of mental illness lead to multisectoral help-seeking, albeit without collaboration between the various disciplines. The CAMH Policy Guidelines do not address traditional service providers or provide a strategy for better integration of services, which might mean continued fragmentation and ineffective service provision of child mental health care.

**Electronic supplementary material:**

The online version of this article (10.1186/s13034-019-0262-7) contains supplementary material, which is available to authorized users.

## Background

Good mental health, especially in childhood, is instrumental to improving health worldwide and has been made a priority on the Sustainable Development Goals agenda [1]. It has been estimated that up to 20% of children and adolescents globally suffer from a debilitating mental illness, and 50% of adult mental illness starts in adolescence [2]. In low and middle income countries (LMICs), 15–30% of the disability-adjusted life years are lost due to neuropsychiatric illness in childhood and adolescence [3, 4]. However, in 2004 it was estimated that up to 85% of people living with a mental illness in LMICs did not receive treatment due to lack of service provision and underutilisation [5, 6].

In Uganda, mental health is recognised as a serious public health problem, and the government has issued policies and legislation with the aim to strengthen mental health care at the primary care level [7]. However, service provision remains a major challenge, and Butabika Hospital in Kampala is the only national referral mental health hospital in the country that includes a children’s ward. Few or none of the mental health outpatient facilities have specialised services for children and adolescents [8] even though this group accounts for 36% of their consultations [9]. Limitations in the general mental health system have been acknowledged in the national Health Sector Strategic Plan III, and underfunding and inadequate staffing and access to medicines, in addition to negative attitudes to the prioritisation of mental health at the managerial level, have been recognised as factors hampering progress in this area [7]. This has resulted in a treatment gap, particularly affecting the child population.

As a response to the inadequate handling of child mental illness, the Ministry of Health recently released the Child and Adolescent Mental Health (CAMH) Policy Guidelines [10]. In these guidelines, factors such as perceptions about childhood behaviour and illness, misinterpretation of these, and limited public knowledge about CAMH are emphasised when explaining the underutilisation of child mental health services [10]. The prior lack of attention to the family as the primary platform for childhood development and well-being is recognised, and improving the knowledge of stakeholders, including parents, is an important objective [10].

In addition to the treatment gap, several studies indicate that there is an epistemological gap in how mental health and illness is conceptualised by the public and by different biomedical and traditional health care providers [11]. A spiritual and supernatural explanation for mental illness, including epilepsy, is long standing in Africa in general [12, 13], including Uganda [14–16]. Religious beliefs and traditional cultural explanations portraying people suffering from mental illness as dangerous, bewitched, or receiving punishment for wrongdoing is common [16]. To ensure an increase in service utilisation, it is crucial that the scaling up process resonates with the users and their caregivers and that the practitioner respects these beliefs [17]. In the case of a patient who is a child, it is the parent’s perceptions of the child’s symptoms, severity, and perceived burden that determine if and where to seek care, and these perceptions are influenced by their knowledge and belief systems [17, 18].

In order to make meaningful improvements in the access to mental health care for children, it is crucial to understand the considerations that parents make regarding mental health and help-seeking [3, 17]. This study explored parents’ perspectives of sociocultural barriers and facilitators in the help-seeking process. More concretely, we investigated how parents recognise a mental health problem in their children, what they believe causes it, and where they would turn to for help. The word ‘parent’ is used here to describe any caregiver or legal primary guardian, whether biological or not. ‘Mental illness’ is used as a term intended to be broader than ‘disorder’, and it adheres more closely to the popular understanding of mental processes and illness.

## Methods

This study was part of the larger research project *SeeTheChild* – *Mental Child Health in Uganda* [19], which aims to estimate the prevalence of childhood mental health conditions and to qualitatively investigate the mental health system from both the user and provider perspective.

### Setting

This study was conducted in the Mbale district in eastern Uganda between July and October 2014. Mbale is not affected by war and consists of urban, semi-urban, and rural areas. Uganda has a population of approximately 35 million people [20] with 54.1% under the age of 18 years [21]. Mbale district has 495,000 inhabitants, and approximately 95,000 live in the urban centre of Mbale Municipality [20]. In 2012, the average household in the Mbale district consisted of 4.4 individuals [22]. In 2011, the under-five mortality rate in the eastern region was 87/1000 live-births [23]. There are 47 health units in the Mbale district, 15 of which are owned by non-governmental organisations, and 78% of the population lives within a 5 km radius of a health unit [22]. At the time of the study, the Mbale regional referral hospital had a psychiatric unit that offered mental health in-patient and out-patient services. There were no trained medical doctor working within the unit, and instead patient care was provided by two clinical health officers holding a diploma in clinical medicine with a specialisation in psychiatry. In addition, the unit had diploma-level nurses and other health workers.

### Study design and procedures

A qualitative study design using focus group discussions (FGDs) was chosen because this is the most appropriate methodology for exploring ideas, concepts, and experiences about a topic in a given cultural context [24, 25]. The treatment gap for childhood mental illness has been established [5, 6], and qualitative investigations to ensure successful implementations of interventions have been called for [3]. FGDs are considered to be particularly sensitive to cultural variables because they open up for discussions about consensus and dissent, allowing for different narratives to unfold and be contested [26]. FGDs are an effective method to gather data and have been shown to stimulate contributions from participants who might be intimidated by individual interviews, especially when discussing sensitive topics [25]. FGDs have also been shown to be suitable for gaining access to community views, which are traditionally accessed by polls, because they allow for community members to express attitudes and to do so in relation to their relevant social context [25]. Assuming most parents have opinions on or experiences with possible symptoms of mental illness in children, we considered FGDs to be a suitable method for exploring the latent reasons for the underutilisation of services.

### Study participants

Parents of children younger than 10 years of age were purposively selected and recruited by mobilisers from the community. Eight FGDs and two pilot group discussions with 6–8 parents were conducted, with a total of 74 participants. The eight groups included two urban and two rural groups of male participants as well as two urban and two rural groups of female participants. ‘Urban area’ was in this context defined as being close to the main municipality in the district and containing a trading centre. Before commencing the FGD, the participants were asked to fill out information about their age, their education level, their main income-generating activity, and the number of children they have and their ages. The age of the participants ranged from 18 to 72 years old, and the number of children ranged from 1 to 9. Thirty-eight were farmers, nine worked within business and sales, seven did manual labour, six did casual labourer, five were house wives, one was a moped cyclist, one was a nurse, one was a teacher and three were unemployed. Four had never attended school, twenty-eight had not finished primary school, three had completed secondary school, and one held a higher education diploma. The purpose of including rural and urban groups was to have a sample of participants from diverse socioeconomic backgrounds as opposed to comparing different groups.

We chose to include only parents who had children younger than 10 years old at the time of data collection in order for them to have a clear view of this age group in mind. Younger children are more dependent on their parents to seek help on their behalf than older children are, and they are less likely to be sought help for because it is commonly believed that the young children will ‘grow out of it’ [27, 28]. Development from childhood to adolescence occurs on an individual continuum, but 10 years is usually considered a critical point of transition [29, 30] with the onset of puberty and its associated hormonal and cognitive changes, combined with a shift from family oriented to peer oriented influences [29].

### Data collection

Two research assistants (one male and one female) with a diploma or bachelor’s degree in the social sciences and experience in qualitative research were responsible for data collection, and they compiled field notes from each FGD and contributed to further development of the topic guide. The research assistants lived in Mbale District and were fluent in all four local vernaculars spoken. One moderated the discussion and the other observed and took notes. Mobilisers recruited relevant participants by asking them to participate and organised a time and place for the FGD. They were community members known to the research team from previous field work in the area, and they were offered a one-time payment for their efforts. Two pilot FGDs were conducted before the formal data collection started in order to assure the relevance and appropriateness of the topic guide in relation to the topic of interest. The first author, VS, observed these pilot FGDs, and it was decided that her presence as a Caucasian was potentially disturbing the discussion, outweighing the benefit of observing the remaining FGDs first hand.

All FGDs were conducted in the participants’ villages or places of residence, and a convenient time and place was agreed upon by the mobiliser and the participants. An appropriate site was arranged by the mobiliser, such as a community hall, an open area, or someone’s home. All participants were informed about the study and its background before the discussion commenced, and they all provided written informed consent. The participants were provided with a transport refund and a small refreshment. Each participant was assigned a letter from A to H for anonymous identification of the speakers in the transcript in order to see whether a viewpoint was shared in the group and whether all participants were active in the discussion. When it was unclear who was speaking, the letter P for participant was assigned. The letter M refers to the moderator. To facilitate discussions, the groups were read a vignette story about a boy exhibiting possible symptoms of mental illness. To ensure representativeness of the views pertaining to more than one set of symptoms, half were told a story about a boy exhibiting symptoms of emotional withdrawal and the other half were told a story about a boy showing signs of hyperactivity. The introductory stories were written by a Norwegian doctor and professor with experience in child and adolescent psychiatry (IE) and a Ugandan child and adolescent psychiatrist (JN) and were based on the DSM IV criteria for depression and ADHD. The stories were recognisable for the participants, confirming that similar children were found in their communities. By using these narratives, the moderator used a topic guide with probing instructions to facilitate group discussion regarding (A) what causes mental health problems in children, (B) what characterises children with mental health problems, (C) what should be done when a child is struggling with mental health issues, and (D) what are the promoting and prohibiting factors for the help-seeking process. The topic guide was translated by a team of local research assistants who were native speakers in the relevant language, Lumasaba (Additional file 1).

The participants were encouraged to speak freely and openly about their knowledge, opinions, and experiences. Each FGD lasted between 1.5 and 2 h and ended when no new issues seemed to arise or when the participants could no longer stay. The moderators noted that there was variation in how openly the groups spoke. The men and the elders spoke more freely than the female and young participants. The moderators probed further when necessary, and they politely asked participants directly for contributions when appropriate in order to ensure a varied contribution and to avoid that the most vocal informants’ perceptions were overrepresented.

All discussions were audio recorded and transcribed by groups of two or three research assistants, directly translating the audio files into English by reaching consensus of the translation because the language spoken in the area is seldom written. To minimise the implications of using translated transcripts, possibly losing some of the original expression of concepts [31], and to ensure the quality of the translation, the audio files and transcripts were listened to and read through a second time by a different group a of research assistants to ensure consensus between the two different groups. The transcripts were discussed with VS in order to evaluate the need for further probing and clarifications on the content. After evaluating the transcripts, no new topics seemed to arise, and we concluded that saturation was achieved.

### Analysis and interpretation

The unit of analysis consisted of the transcripts from the FGDs and pilot FGDs along with field notes. The transcripts were transferred to the open coding software NVIVO 10 for initial data sorting and analysis. The raw data were thoroughly read in full text by VS and IE to gain a sense of the whole before VS further coded the meaning units and sorted the data according to Graneheim and Lundman’s framework for content analysis, excluding the condensation process [32]. This framework was chosen because it provides a comprehensive framework for content analysis as well as evaluation of trustworthiness, and it focuses on context, which is warranted when exploring culturally sensitive topics. Being explorative in nature, the analysis was done with a bottom to top approach in which the smallest meaning-bearing units were coded and systematically put into categories and sub-categories, and eventually into themes emerging across categories. For example, the text *P1: If he is a person with a mental problem there is nothing doctors can do. They first give medicine to calm him down, then examine him, and if he is found to have a mental illness, he is then taken to ‘a room for mad people’. M: Where else do you take a person with mental health problem? P2: Witch doctors. [Murmuring from participants] P3: People prefer to be prayed for. M: To churches? P2: Yes, we think there are demons disturbing him* was grouped into the category ‘where to seek help’, and the first part was grouped into the subcategory ‘doctor for calming down’ and the second part was grouped into the subcategory ‘pastor and witch doctor for handling the spirits’, both of which consequently became part of the intercategorical theme ‘a web of beliefs about causes results in multisectoral help-seeking’.

The crude coding tree was presented and discussed with AA, JN, IE, and VS to ensure representativeness of the findings. The coding was done by VS; however, the coding process and the abstraction of themes was closely consulted and discussed with the research assistants and researchers to ensure the validity of the analysis and the trustworthiness of the findings. After the coding and themes were agreed upon, the raw transcripts were read again in full text by VS to verify that the themes that were identified reflected the overall impression of the data.

### Trustworthiness of the findings

The credibility, dependability, and transferability of the analysis and results was assessed using Graneheim and Lundman’s measures for achieving trustworthiness [32]. We composed groups with varied insights and experiences regarding the research topic in order to facilitate discussion and to have a broad range of viewpoints represented. This was done by organising groups with diverse socioeconomic backgrounds and having both genders represented. We decided to divide the groups into men and women due to methodological considerations of group dynamics, where we sought to have the groups be homogenous enough to ensure free expression but diverse enough to encourage a rich discussion [26]. Additionally, it was suggested by the Ugandan research assistants that Uganda is a patriarchal society and that women might hesitate to disagree or to voice their opinion with men present. The team of researchers involved in the analysis had varied academic expertise with both local context-specific and external perspectives, and this contributed to a nuanced discussion and interpretation of the data. The participants’ recognition of the findings was not sought because this was difficult to obtain in practice. We appreciate that norms, perceptions, and practices might change over time, especially in communities undergoing rapid development and change. However, the perceptions and practices we were exploring were deemed by the team to likely be consistent within the context and to remain relevant over time. Mbale district has not been affected by war, and there are no major vulnerability factors in the community that are unique to the area. We therefore believe that the findings are transferable to similar settings.

## Results

The participants first discussed the introductory vignettes about a hyperactive or depressed child and thereafter discussed mental health in general. From the analysis, the following themes were identified and will be further elaborated on in the following section: (1) a distinction between a challenging and a mentally ill child, (2) a web of beliefs about causes results in multisectoral help-seeking, and (3) weakened social support structures hamper access to care. When recognising mental illness, there was a distinction between the stubborn and the ill child, where the latter required a rather serious symptom load to be considered ill. There was an overlap between spiritual, biomedical, and psychosocial explanations for symptoms, causes, and treatments, and there was no mutual exclusiveness between these paradigms, making multiple help providers relevant simultaneously or consecutively. A loss of social support structures in the community affected opportunities for help-seeking when an ill child was recognised.

### Stubborn or ill? Recognising mental illness

The participants reported various symptoms as relevant when recognising a potential mental health problem or illness in their child. There was a certain distinction between what they perceived as abnormal behaviour and what they deemed as mental illness, although these could share a common cause. Mental illness was spontaneously described with severe or psychosis-like symptoms, representing a clear breach of normality. Softer symptoms, such as being restless or not playful, were always regarded as abnormal and worrisome, but it varied as to whether these were considered a sign of mental illness. In general, mental health problems were described with visible symptoms and behaviours rather than thoughts and emotions. Worry arose from a noticeable change in the child, concern for the child’s well-being, and the fear of stigma.

#### The stubborn child

The term ‘stubborn’ was often used when describing and discussing the vignettes about the children expressing symptoms of depression or ADHD. A stubborn child could be both quiet and active, but did not act as expected or respond well to disciplinary efforts. A stubborn child was a challenging and not entirely normal child, but was not necessarily an ill child.

While discussing the depressed boy, sleeplessness, not eating, and having unsettled thoughts were recognised as abnormal and were considered signs that the child was having a problem. It was emphasised as worrisome if the child exhibited an unwillingness to play. Playing and a wish to socialise with other children was an important sign of childhood wellbeing, and it was considered alarming if the child did not play. Other signs and symptoms could be present, but when the child did not want to play, one knew the child was struggling. A playful and social child was a healthy child. One father explained:

*C: For me as I heard that story, it gives me worries. The reason being, I don’t know what has hurt his body. Because instructing a child to go to school and he instead gets worried is surprising. You tell him to go and play with friends and he instead sits on the veranda that worries me even more [FGD 7, rural fathers].*

When discussing the hyperactive child, concerns included restlessness, stubbornness, disturbing others, and fear of the child destroying other people’s property. The concern was not mainly expressed relating to the child’s well-being, but rather that having an unruly child in a close community and school setting might risk social sanctions, embarrassment, and stigma. However, hyperactivity was not unanimously seen as a problem. A child full of energy was by some considered to be a resource and was mentioned as a sign of brightness and creativity. One father explained that *it means he is so bright and in the future is going to be useful and helpful to the community. As a parent you have to think of a way to model his brilliance even if it means investing money in him [FGD 8, urban fathers].*

Although stubbornness was not considered to be mental illness, a noticeable change in the child’s stubbornness was an important cause for worry. The concerns arose from fear of the child being unruly and disrupting community life and risking social sanctions and embarrassment. Another important worry was the fear of unmet expectations about future success and helping the family. The child was expected to contribute to the family, and the energy put into child rearing and education was expected to yield outcomes; *P4: Yes I become worried about him because when you produce a child and you take him to school, you expect him to help you in the future. But when he refuses to go to school, it is as if he is useless [FGD1, rural mothers].*

As this mother expressed, a stubborn child, either unruly or passive, might be unable to meet the expectation of future help and support to the family and as such might be considered useless and as a disappointment.

#### The mentally ill child

When discussing mental illness in general, the symptom description differed vastly from the concerns related to the vignette stories. Unacceptable psychosis-like behaviour was spontaneously shared when discussing how a mentally ill person behaved, and epilepsy was an important, but not well-defined ailment, as explained with various symptom descriptions.

Symptoms of mental illness, such as undressing, smearing faeces, eating from dustbins, and being violent, were readily shared. *E: Mental health problems are when you meet a child on the way and he begins laughing, picking up things from the street or even before you begin talking to him, he begins laughing […]D: You know that a person has mental health problems when you see the person strip and smear faeces and you just know [FGD 1, rural mothers].* Serious symptom descriptions like these were more spontaneously expressed compared to the symptomatology in the vignette stories, and these seemed closer to their general concept of mental illness. The parents also expressed a fear of people with these symptoms because they were deemed unpredictable and a potential source of harm and insecurity in the community.

The term ‘epilepsy’ encompassed a wide range of symptoms not necessarily related to the neurological understanding of the illness. The term was tightly connected to the concept of mental illness; however, there was no clear consensus on epilepsy and epileptic seizures as a characteristic of mental illness. It was said to be a mental illness, a cause for mental illness, and/or a differential diagnosis. It was always used as a symptom or description of something being wrong, but the individual using the word attributed different meanings to it. Symptoms of epilepsy ranged from ‘*tsifubu*’, meaning convulsions and fit, to disobedience; *P: The way he is disobedient, he could be having a mental problem. M: Is that an illness? P: Yes…because when you tell him something, he does not understand it. M: What could that illness be? (…) D: Epilepsy [FGD 4, urban mothers]*. Having a child with ‘epilepsy’ was a source of stigma; *F: If you have a child with epilepsy, you will fear mentioning it in public. […]because people think when you get near an epileptic person you will get infected with it, too [FGD2 rural fathers].* However, some participants spoke up against these stigmatising beliefs and stated that people with epilepsy can live normal lives.

### Finding the cause—a web of spirits, imbalance, and spoiling

The onset and development of mental illness was seen as multifactorial, with no mutual exclusiveness between biomedical, psychosocial, and spiritual explanations. Mental illness in childhood could be inborn, inherited from the parents, or passed on through spirits.

#### Ancestors, spirits, and witchcraft

Supernatural causes were an important and widely shared explanation for symptoms of mental illness, including epilepsy. Attacks by spirits and demons, ancestral spirits, and witchcraft could be inborn or could affect a person later in life. These spirits could be part of a clan, be a punishment from God, a spell cast through witchcraft, or could be passed on through ancestors. In particular, being named after an ancestor was a pathway for a child to be affected by the late person’s characteristics, including mental health symptoms. To avoid this, the elders could be consulted on what to name a child to prevent certain traits, or they could help a child get rid of the spirits by calling the names and performing rituals. One of the parents explained how elders might be consulted:

*D: Sometimes it could be a clan issue. The naming of the child involves the coming together of grandparents of both the father and mother of the child, preparing a dinner, and then calling on all the names of the ancestral spirit (…)the elders will mobilize contributions from the community and advise you to make a contribution of either local brew or a chicken or a goat. The elders know the names of the spirits and their behaviours when they were still alive and know if such names can be carried forward or not [FGD1, rural mothers].*

#### Loose wires and imbalances

Although the spiritual aspect was emphasised, there was a clear belief that the brain could be the source of mental illness. It was believed that diseases such as cerebral malaria or medicines like quinine and contraceptive pills might affect the brain of the child and cause mental illness. A balanced brain was a healthy brain, and disease, medicines, blood volume, and alignment of ‘wires’, meaning nerve cells, might disturb this balance and cause symptoms. Having too much blood, dysfunctional veins, or ‘wires’ could make the brain ‘uncoordinated’, and wires could become loose and disconnected. One participant explained how malaria could disturb the wires:

*P: That child might have suffered from malaria in his childhood. Therefore he has two wires that are disconnected, so whenever they meet again that child starts behaving weird. But if they do not meet and spark, he is fine [Pilot FGD, fathers].*

The following exchange between the moderator and the participants illustrates the traditional belief in blood flow as a source of abnormal behaviour:

*M: Ancient people knew many things; if their cow would jump up and down they would say it is because it has excess blood. P: They would strike a vein and blood would flow and the cow would stop disturbing them. E: This child is different because in his brain there could be one or two veins that are not working [FGD 7, rural fathers].*

#### To spoil a child

The parents discussed how parents could ‘spoil’ a child, and from the transcripts, and from discussion with the research assistants, it seems that ‘spoil’ is used as a synonym for ‘ruin’. Parents could spoil, or ruin, their child mainly through parenting, which could affect the child’s mental health and development, most notably through disciplining strategies such as corporal punishment. It was recognised by some that corporal punishment and lack of care from parents and teachers could be a perceived negatively by the child, making them stubborn or driving them away from home. However, this was said to be a common form of disciplining, and there was substantial disagreement as to whether corporal punishment would spoil or help the child.

#### Substance abuse

Another environmental factor that could affect the child was if they used alcohol or other substances. Being drunk was considered by some as a cause for mental illness and by others as an alternative cause for symptoms that should be excluded before seeking help by letting the person become sober. Children were also exposed to substance use, which could cause and worsen mental illness in childhood:

*E: His brain is too weak and it gets worse when taking [drinking] alcohol.(…) A child like Joshua [the hyperactive boy in vignette story] can be restored, but here in our community, being a slum, it’s difficult due to the high population and a lot of drug abuse. M: Is he different from other children? E: Yes……when he starts using drugs, he becomes different. M: Do all children here take drugs? Ps: Some do and some do not [FGD3, urban fathers].*

This was, however, a minor theme, not commonly reported, but with possible substantial implications.

### Finding help—addressing all the causes

When the parents discussed where to go if they were worried, or if a mental illness was suspected, doctors, religious leaders, elders, traditional leaders, witch doctors, teachers, peers, and the legal authorities were all considered relevant help providers. Multiple providers could be approached in parallel because the expected treatment varied.

#### Handling the spirits

A spiritual component was perceived by most to be essential to successful treatment because it targeted what was believed to be the underlying cause of the symptoms. Elders, religious leaders, traditional healers, and witch doctors were equally qualified to be consulted instead of, before, or in parallel with medical personnel. Rituals, sacrificing animals to ancestors, or praying for the patient were important measures depending on the believed cause. When a child had received traits and characteristics by being named after an ancestor, this could be handled as explained: *A: They take you back to the clan and they slaughter chickens in the shrines that they have built (…) They call the names of those old people who died long ago, and the person gets well [FGD 5, rural mothers].* Because these help providers were widely accepted and accessible, they were often consulted to see if the child would get better before going to a health centre.

#### Injections for calming down

Health facilities were mostly expected to examine the patient, medically relieve symptoms, provide advice, and produce a report for other help providers in further handling the patient. Given that mental illness was considered to be associated with psychosis-like behaviour, injections and medicines to ‘calm the patient down’ were expected to be provided by health facilities when seeking treatment. This was, in addition to confinement, important to making the patient more cooperative and amenable to treatment elsewhere by religious leaders or by more specialised care units. There was no strongly expressed belief that health workers could heal mental illness, except one participant who mentioned therapeutic counselling by medical personnel: *E: The treatment is of several kinds, when a person has just started exhibiting the symptoms, there are doctors for the brain. They can treat him just by talking to him or just by asking him questions, and he gets healed [FDG 8, urban fathers].*

#### Teachers as co-parents

Most participants considered teachers to be a key source of information on how the child behaved and how unwanted behaviour could be changed. The teacher could be consulted before going to the help provider of choice, and the teacher would receive the report after the examination and would contribute to the management of the child. They were regarded as an important observer of the child and a key disciplinarian; *P: A teacher is also a parent and can control someone and he grows up well behaved. If he is stubborn and you handle him with a heavy hand, you might spoil him more. But the teachers know how to do it, and they discipline him in a simpler way [Pilot FGD, fathers].* It varied as to whether the parents expected more or less corporal punishment for disciplining in school because this was viewed as both helpful and harmful. Juvenile detention centres, called remand homes, were considered to be an appropriate help provider by some, especially for behavioural problems. However, they were seen as a last resort when everything else had failed and the problematic conduct persisted.

### Changing social structures influence help-seeking

A more subtle theme was how changes in the way the community handled children led to insecurity regarding the possibility to help others, as well as what help one could expect for one’s own children. Some reported a loss in collective responsibility for observation of other people’s children, which hampered the recognition and handling of symptoms associated with mental illness. The parents shared that in the past it was expected of parents to share observations and provide advice regarding children in the community, a practice now considered unwanted interference. A father explained; *G: (…) These days, you might discover your neighbour’s child has not gone to school, but when you beat him to get him to go to school, the child reports you to his father. The father will come and quarrel with you, saying: “How can you beat my child? Are you the one feeding him?” [FGD 8, urban fathers].* This was reported by some as a change from the traditional community handling of child rearing and disciplining into a more individualistic matter that was exclusive to the nuclear family.

This shift in responsibility also affected the expected possibility to obtain appropriate help due to a lack of financial and practical support. Most of the participants reported that finding money and time for treatment and transportation were considered substantial barriers to seeking help, for which one could previously turn to the community for advice and practical support.

## Discussion

This study explored community parents’ perspectives regarding child mental health from the recognition of symptoms to help-seeking. The following three main themes arose: (1) a distinction between a challenging and mentally ill child, (2) a web of beliefs about causes results in multisectoral help seeking, and (3) weakened social support structures hamper access to care. Descriptions of severe symptoms and epileptic seizures (including unconventional understandings of epilepsy) in clear breach with normal behaviour were emphasised when recognising mental illness, while symptoms of common mental illnesses, such as not wanting to play and destroying other people’s property, were disregarded as stubbornness and not needing treatment. A mixture of supernatural, biomedical, and environmental understandings of the causes of these symptoms were reflected in a complex pattern of help-seeking, where treatment providers such as traditional healers, witch doctors, medical doctors and religious leaders were contacted simultaneously. There was disagreement as to whether environmental factors such as corporal punishment were beneficial for discipline or were risk factors for mental illness. A finding that should be further explored is the notion that a loss of social structures in the community seems to hamper the recognition of vulnerable children and their access to care.

### A challenging or an ill child?

Mental health problems were mostly described with visible symptoms and behaviours, as opposed to thoughts and emotions. When discussing mental illness, the participants shared descriptions of visibly aberrant behaviour, such as undressing and having fits, and thus representing a clear breach with normality. This seemed closer to their spontaneous understanding of mental illness compared to the softer symptoms of depression and hyperactivity, which were recognised as a problem but were to a lesser degree seen as a mental illness. The lack of parental recognition of common mental illnesses in children can be attributed to the cultural understanding of normality and how symptoms affect daily living, conceptualised as the ‘perceived parental burden’ by Costello et al. [33].

Symptoms of common mental illnesses, such as depression and anxiety, represent behaviour on one end of a spectrum of normality rather than a clear disruption from normality. Findings in research on adult mental health in Uganda show that common mental illnesses like anxiety and depression are attributed by lay people to ‘thinking too much’ [15] and that there is the notion that these symptoms are not necessarily linked to a mental illness but are merely a part of life [16]. The line between normality and mental illness is not clearly defined, leading to variation across ethnic and cultural groups as to what qualifies as mental illness and how it is appropriately handled [34, 35].

In our findings, having a ‘stubborn’ child was a source of worry, and the level of worry has previously been found to predict the initiation of the help-seeking process on behalf of children [27]. However, in the framework for child mental help-seeking called ‘The Children’s Network Episode Model’ (Children’s NEM), the concept of ‘perceived parental burden’ is argued to be a stronger predictor for help-seeking compared to worry [33]. Parental burden correlates with how the symptom load affects perceived impairment and interference with daily living. Although worrying can be burdensome in itself, help-seeking is more often preceded by symptoms that incur stigma or social sanctions or that make it difficult to carry out everyday chores [33, 36]. Extrovert, disruptive, and notably abnormal behaviours in children, as emphasised by our participants, have also been shown to promote help-seeking [37, 38] compared to introverted symptoms such as an unwillingness to play [37, 39], and this might be due to differing impacts on perceived parental burden [33, 36].

The link between the perception of normality and the perceived parental burden and having a child with clearly abnormal and urgent symptoms can help explain why symptoms such as seizures and undressing publicly were emphasised when describing mental illness. Especially striking is how this is reflected in data from supervision reports showing that epilepsy and neurological diseases account for 75% of the mental health consultations in Uganda [7]. However, the lack of recognition of symptoms on the spectrum of normality has been shown to leave common mental illnesses such as depression and anxiety unattended [40]. Increased knowledge about mental health in the general community is recognised as a key aim for the CAMH Policy Guidelines [10] because leaving these unattended will have far-reaching public health consequences [41].

### The need for multisectoral collaboration

The participants reported a diverse range of causes for mental illness. A supernatural component of mental illness, including epilepsy, is well known in Uganda [14–16] and throughout much of the African continent [12, 13]. The imbalances in blood volume and misaligned ‘wires’ resembles a humoral understanding of illness dating back to the ancient Greeks [42], and such an understanding has been reported by 15% of Indians as the cause of their psychiatric illness [43]. Corporal punishment in the home and at school was regarded as important, albeit with a certain disagreement as to whether it would help or ‘spoil’ the child. School violence has been connected to poor mental health outcomes [44], and the promotion of children’s rights and protection against corporal punishment and physical abuse has in recent years been advocated in Uganda and elsewhere in Africa [45]. A minor but potentially important finding was childhood drinking. Substance use before age 10 has been described in LMICs [46–48] and might be of significance because Uganda has one of the world’s highest rates of alcohol consumption [49].

The multifactorial character of the causes of abnormal behaviour was reflected in the help-seeking process. Parents reported that they would rarely seek advice or help from only one care provider, resulting in medical professionals, teachers, religious leaders, and traditional healers being consulted simultaneously or in different phases of the illness. The school was regarded as a key place for recognition, management, and advice relating to child mental illness, and there was a strong sense of trust in teachers and their competence in disciplining and changing unwanted behaviour. There was no consistent hierarchy in the sequence of where to go first, and help providers were considered relevant for different aspects of the illness. As an example, extrovert or aggressive behaviour might be explained by spirits, but a medical doctor might be helpful by calming the symptoms, albeit not necessarily targeting the issue that was believed to be the root cause. In this sense, the different institutions complemented each other and worked as ‘parallel health systems’, and such a phenomenon has been observed elsewhere in Africa [11, 17, 50, 51]. In 2015, Burns et al. conducted a systematic review and meta-analysis of the use of traditional and religious healers in the pathway to care for people with mental illness in Africa and found that 48.1% consulted a traditional or religious leader first [50]. In their review, only one study focused on children, but it reported similar findings of help-seeking from a complex web of institutions including school, family, and both formal and informal health systems [52].

As urged by Burns et al., the multimodal use of the health system should be taken into account by the help providers, and collaboration between providers should be strengthened [50]. In Uganda, Abbo et al. found that patients who combined care from the traditional and biomedical health systems had better outcomes, and their study concluded that stronger collaboration is imperative for improved mental health care [53]. However, unlike the users of health care, help providers have been shown to have a clear sense of identity and mutual distrust [11], potentially hampering the possibilities for collaboration [11, 54]. Unfortunately this is reflected in the CAMH Policy Guidelines where the spiritual basis for understanding mental illness and a potential collaboration with other help providers is not discussed or addressed [10], possibly maintaining a gap in understanding between the users and providers of health care in Uganda [17].

A point where the participants and the CAMH Policy Guidelines do agree was the emphasised importance of the school [10]. The function of the school has been recognised as a distinct component of child mental help-seeking [33], and it acts in the CAMH Policy Guidelines as an important measure to improve the accessibility and availability of services [10]. This common understanding between the parents and help providers might suggest that the school is a good arena for improved mental health focus and collaboration between different sectors. It might also be a relevant arena for preventative measures and for recognition of children at risk for harmful substance use. The notion that young children in families might be using alcohol and other substances is also recognised in the CAMH Policy Guidelines, stating that *“alcohol and drug abuse in children and adolescents in Uganda is on the increase although not well researched”* [10].

### A changing community

The notion of loss of social support structures in the community is a finding that should be further explored because it might significantly influence the help-seeking process. Our findings were not comprehensive enough to draw conclusions from this, but our participants noted a change towards a more exclusive handling of family affairs, leading to restrictions on reporting and disciplining other’s children and a fear of not receiving practical support from others. African societies are traditionally characterised by collective community efforts for social security [15, 17], which has been suggested to promote resilience against mental illness [17]. It is worth noting that the reported change has been recognised in the CAMH Policy Guidelines, suggesting that “*weakening family and social support structures*” might be a threat to the aim of increasing knowledge and involvement of, among others, families and community leaders. This loss of reliable informal social security and collective effort for child rearing might delay the process of symptom recognition and help-seeking, leaving a vacuum that must be addressed and replaced by the public health system.

### Policy recommendations

Our findings confirm some of the policy priorities outlined in the CAMH Policy Guidelines and establishes some new ones. In order to improve the outcomes of children and young people suffering from mental ill health in Ugandan communities, several steps must be taken. Continued focus on misconceptions about causes must be addressed to reduce stigma and promote help-seeking. Increased awareness about symptoms closer to normal behaviour must be prioritised to improve recognition of common mental illness in children. The recognition of young children possibly using alcohol and other substances must be further explored and appropriately managed.

Stakeholders should capitalise on the common recognition of the importance of the school when planning the upscaling of and improved access to services. Teachers and parents must be sensitised to the importance of mental health in children, the symptoms of mental illness, and the opportunities for seeking help. The recognised weakening of informal social security networks traditionally provided by the community warrants an appropriate response to replace this with formal public services.

The CAMH Policy Guidelines do not address traditional service providers or provide a strategy for better integration of services, and this might facilitate continued fragmentation and ineffective service provision of child mental health care. The formal health system has to resonate with the users, and it must respect the widespread belief in the supernatural aspect of mental illness while ensuring access to evidence-based medical care. There should be a recognition of the multifactorial beliefs about the causes of mental ill health that lead to multisectoral help-seeking. The various help providers must strive to collaborate despite their differences in beliefs, appreciating that service users do not perceive them to be mutually exclusive and prefer consulting them simultaneously.

### Limitations and methodological concerns

To facilitate a healthy group dynamic and a safe environment for sharing, we tried to make the groups relatively internally homogenous with respect to gender and socioeconomic status [26]. However, the large variation in age might have contributed to socially desirable answers across generations. Because we prioritised diversity between the groups in order to have a varied set of participants, relatively few people ended up representing each group (rural/urban and male/female). However, the study was not designed to make valid comparisons between the groups. None of the parents expressed their own experiences with help-seeking for their own children, thus there might be a discrepancy between what they would do in theory and in practice. Although FGDs have been shown to also work well when discussing sensitive topics [25], they are not as suitable as in-depth interviews for accessing personal experiences. For discretion, we did not ask the parents directly to share experiences with mental illness in their own children, and no one shared this information spontaneously. The discussion focused on children in general, and not on one specific gender. However, because both vignettes were about a boy child, this might have influenced the participants’ considerations. Although it is an interesting topic, it was beyond the scope of the study to assess gender differences in the evaluation of mental illness in children. We acknowledge the limitation in using translated transcripts, possibly losing some of the original expression of concepts. However, we tried to minimise the impact this had on the analysis by using bilingual research assistants who had both the local language and English as their native language and having two groups of research assistants reach consensus on the translation. The research assistants were also involved when discussing the content and analysis of the translations [31].

The data were coded by VS, but agreement on the findings and their representativeness was sought within the team of researchers with expertise in both the context and methods in order to strengthen the trustworthiness of the analysis. The data were collected by VS as part of her doctoral study. The process of analysis and interpretation of the findings might have been influenced by VS’s background as a Norwegian medical student, coming from a different health system and culture in which awareness and access to child mental health care is widespread and where supernatural causes are not commonly considered. Throughout the research process, VS has been highly aware of her own position as an outsider and has tried to remain open to the local beliefs and context. She spent 8 months in Uganda doing fieldwork and clinical rotations in paediatric and child and adolescent psychiatry at Makerere University in Kampala, Uganda. She continuously consulted the research team from Uganda in order to minimise the impact of obvious differences within her own and the relevant context and to address the latent and relevant information useful for the purpose of the study.

## Conclusion

This article shows that there is a discrepancy between how parents and the formal health system, as presented in the CAMH Policy Guidelines, evaluate and handle symptoms and mental illness in Uganda. We suggest that increased awareness of common mental illnesses closer to normal behaviour should be prioritised in order to help bridge the treatment gap. Attention should also be paid to the notion that children might be using substances. We argue that a common recognition of schools as a trusted arena by both parents and policymakers should be capitalised on, and teachers should be trained to recognise symptoms and to promote mental well-being. The reported sense of weakened social security in the communities should be further explored because this might represent a significant barrier to help-seeking. The current CAMH Policy Guidelines lack any recognition of the informal health system. With many service users citing the simultaneous use of various help providers with differently perceived aetiologies of mental ill-health, it is essential that these various help providers are able to ensure cohesive care to the benefit of the patient, and this will require novel and integrated approaches.

## Additional file

**Additional file 1.** Focus group discussion topic guide.

## Abbreviations

CAMH: Child and Adolescent Mental Health
FGD: focus group discussion
LMIC: low and middle income country
The Children’s NEM: The Children’s Network Episode Model
