# Supplementary material for: Child mental illness and the help-seeking process: a qualitative study among parents in a Ugandan community
Source: Child Adolesc Psychiatry Ment Health. 2019 Jan 11;13:3. doi: 10.1186/s13034-019-0262-7 (PMC6329129; doi:10.1186/s13034-019-0262-7)
Supplement: Supplementary file 1 — Additional file 1. Focus group discussion topic guide. [file 13034_2019_262_MOESM1_ESM.docx]

Focus group discussion topic guide

*Introduction:*

Thank you for accepting to participating in this discussion. We all care for our children and the purpose of this discussion is to better understand issues which are relevant for our children. You can choose if you talk about your own experiences or if you talk about issues you know about. We all agree that whatever said is only shared within this group. All the information you share will only be used for research purposes and will be kept anonymous.

Our topic as you know is mental health of our children. First I would like you to listen to a story about a child.

*Vignette 1:*

Little Joshua has just started school and he is 7 years old. He has an older sister and two younger siblings. The older sister has been in school for four years. She is already a fairly good reader. She helps her grandma reading the newspaper as her grandma has problems seeing well. The entire family has always appreciated school and prioritised that. Paying school-fees has always been at the heart of their priorities. Joshua has been different from his older sister in many ways. He has been more restless struggling to fall asleep. In many ways he has been wilder while playing and also maybe a bit clumsy. The parents and family members have thought that this is only because he is a boy. Recently he has started being more disobedient too. He is more restless, disobedient and sleeps less than other boys in the family (cousins, neighbours). The teacher has also indicated that she has problems with him in the classroom and that he either disturbs others or seems to lose focus completely. If you are strict with Joshua you can see he is really struggling to keep calm for a while, before he easily gets restless again and start to distract others.

*Vignette 2:*

Little Francis turned 7 last Christmas; everybody in the big family remembers his birthday because it happened Christmas night. Francis’ mother had noticed a change in Francis over the last three months. Before he showed some interest for his siblings and playing with the neighbour boys, however, he seldom took the lead or initiative for anything. Now his initiative is getting less and less, when something is really funny he smiles a little, but you seldom hear him laughing like the other children. He often withdraws back and sits alone. Francis has not had more malaria than the other children and has never been hospitalised with malaria, the medication sold at the dispensary has always been enough. The kids in the family share some bed nets, and they are fairly well covered. Francis’ mum thought he maybe was anaemic and took him to a clinician for a check-up who found nothing wrong with him. The clinician told the boy just has to just continue with his school as usual. Now Francis seems very tired in the morning and find excuses for not going to school. He finds no joy in what he is doing and get annoyed if he is asked to assist at home. If his younger siblings wants to play with him he gives them irritated comments. Even if Francis is tired in the morning, he has difficulty falling asleep at night. He has lost his appetite, but not yet lost weight to a visible degree. Francis’ dad thinks he is lazy and is annoyed with the mother being worried about Francis. Actually, the mum and dad have recently been quarrelling behind closed doors and even had fights. They have had major misunderstandings regarding the household economy and potential other partners, but they think the kids are uninformed about this. They have both tried to hide their misunderstandings from the children.

1. Would you be worried about Joshua/Francis?
2. Would you think that Joshua/Francis is very different from most boys of his age?

*(Probe for how and why)*

1. Would you think Joshua might have a condition making him sleepless or restless/sad and uninterested?
2. Would you think Joshua/Francis should be discussed with the teacher or any other health personnel? Please discuss.
3. Do you know about other kids resembling Joshua/Francis? How are these type of issues usually handled in your community?
4. Do you have any other suggestions to how to handle these types of difficulties described for Joshua/Francis?

Thank you for your reflections. Below I would like us to discuss the following issues relevant for children only.

*In the next section: Probe for*

- *Conditions (symptoms), severity, duration, reduction in function*

*Remember: This section is about mental health problems in general*

1. What is a mental health problem?

*(Probe for difficulties related to thinking, learning, relationships, behaviour. Probe for symptoms, diagnosis, causes, explanations, reasons)*

1. When can one seek help from the health system?

*(Probe for symptom severity, symptoms, conditions, duration, reduction in function)*

1. How and for what can the health system contribute with help for mental health problems?

*(Probe for difficulties related to thinking, learning, relationships, behaviour. Probe for symptoms, diagnosis, explanations, reasons)*

1. Where else can one expect to get help?
2. How is the access to health systems which could support if one was suffering from a mental health condition? What would be the different services for adults and children? *(Probe for: Where they can go, facilities they know about, if it is easy or hard to access)*
3. How is the social community influencing a care-taker’s ability to seek help from the health system in cases like Joshua, or for other conditions you have been mentioning?
   *(Probe for positive AND negative influence. Barriers, stigma, awareness)*
4. What type of resources would a care-taker need in order to approach the health system with these types of issues?
